# 抑癌基因*TCF21*对肺癌细胞A549增殖、凋亡和迁移的影响

**DOI:** 10.3779/j.issn.1009-3419.2014.04.03

**Published:** 2014-04-20

**Authors:** 松 胡, 诺 阳, 铭伍 陈, 建极 郭, 磊 冼

**Affiliations:** 530021 南宁，广西医科大学第一附属医院心胸外科 Department of Cardiothoracic Surgery, the First Affiliated Hospital of Guangxi Medical University, Nanning 530021, China

**Keywords:** A549, 抑癌基因, TCF21, 凋亡, 迁移, 细胞增殖, A549, Tumor suppressor, Transcription factor 21, Apoptosis, Cell proliferation

## Abstract

**背景与目的:**

转录因子21（transcription factor 21, *TCF21*）是新近发现的抑癌基因，其在多种肿瘤中具有抑癌功能，本研究旨在探讨*TCF21*基因对人肺癌A549细胞增殖、迁移和凋亡的影响。

**方法:**

利用慢病毒转染技术在肺癌A549细胞中高表达*TCF21*基因，以荧光定量PCR、Western blot分析目的基因的表达，并采用Transwell、MTT法、流式细胞术检测TCF21高表达对A549迁移、增殖、凋亡的影响。

**结果:**

成功在肺癌细胞株A549中高表达TCF21，且高表达TCF21后A549的细胞生长和迁移能力受抑制、凋亡率增高。

**结论:**

抑癌基因*TCF21*可抑制A549细胞的增殖和迁移、诱导凋亡。

## Abstract (en)

**Background and objective:**

*TCF21*, a newly discovered gene, exhibits tumor suppressor function in a variety of tumors. This study aims to observe the effects of TCF21 on the proliferation, apoptosis and migration of A549 human lung adenocarcinoma epithelial cells.

**Methods:**

TCF21 was overexpressed in A549 cells via lentiviral transfection. Fluorescence-based quantitative polymerase chain reaction and Western blot analysis were used to analyze the expression of the target gene. Transwell, proliferation assay, and flow cytometry were applied to detect the effect of TCF21 overexpression on the migration, proliferation, and apoptosis of A549 cells after transfection.

**Results:**

The proliferation and migration of A549 cells were inhibited, and the apoptotic rate was increased by overexpressing TCF21.

**Conclusion:**

The tumor suppressor gene, *TCF21*, significantly inhibits the proliferation and migration, as well as facilitates early apoptosis of A549 cells.

肺癌是临床常见恶性肿瘤，并且有着最高的癌症相关死亡率^[1]^。非小细胞肺癌（non-small cell lung cancer, NSCLC）是最常见的肺癌类型，约占到了肺癌的85%^[2]^。由于75%的肺癌患者在确诊时已经有局部或者远处转移，因此肺癌患者总的5年生存率非常低^[3]^。对肺癌发生的可能分子机制研究有限，是肺癌早期诊断率低和患者预后不良的重要原因之一。

抑癌基因的失活会导致肿瘤的形成和恶性转化。目前，许多研究^[4-10]^表明，抑癌基因的失活在肺癌的发生发展中扮演着重要角色。转录因子21（transcription factor 21, *TCF21*）是一个新发现的抑癌基因，它在多种肿瘤中因存在启动子区甲基化，而导致TCF21在癌组织中的表达较癌旁组织降低^[11-13]^。前期研究^[14]^表明TCF21在肺癌中低表达，这与其启动子区甲基化程度升高有关。而这种表达降低对肺癌的发生发展有何意义，目前尚无相关报道。

慢病毒载体可以整合、携带目的基因，并侵染靶细胞，利用这一技术可以在靶细胞中大量表达目的基因。使用携带有绿色荧光蛋白基因的慢病毒转染细胞，可以通过观察绿色荧光蛋白的表达来初步判断慢病毒转染细胞的转染效率。基于本课题组前期组织水平的研究结果，本研究从细胞水平探索TCF21的生物学功能。利用慢病毒转染技术在肺癌细胞A549中高表达TCF21，用荧光定量PCR和Western blot技术检测*TCF21*基因的mRNA和蛋白的表达水平。在确定高表达成功后，利用MTT实验、Transwell、流式细胞术检测TCF21过表达对肺癌细胞增殖、迁移、凋亡的影响。

## 1 材料与方法

### 1.1 细胞培养

人肺癌细胞A549购自中科院上海细胞库，用含10%胎牛血清，1%的青霉素链霉素的1640培养基，于37 ℃、5% CO_2_条件下培养。

### 1.2 慢病毒转染

过表达慢病毒载体由Invitrogen公司合成并构建。细胞分为三组：第一组为转染Lenti-TCF21组（过表达组），第二组为转染空载体病毒组（空白载体组），第三组为不转染病毒组（未处理组）。转染流程按照Invitrogen公司说明书进行。于转染前8 h将A549细胞按3, 000个/孔接种至24孔板，待细胞融合率达到30%-50%时开始转染。转染MOI值为100，总液体量为500 μL/孔。转染10 h后将液体换为含10%胎牛血清的1640培养基继续培养。在转染后12 h、24 h、36 h、48 h、60 h、72 h、84 h、96 h共8个时点，随机选取多个视野，分别计数白光与荧光条件下的可见细胞数，通过相同视野中荧光条件下可见细胞数与白光下可见细胞数之比来计算平均转染效率。转染72 h后荧光最强，于此时收获细胞用于下一步实验。

### 1.3 荧光定量PCR

按照RNA提取试剂盒说明书步骤提取细胞总RNA。然后使用逆转录试剂盒将其逆转录为cDNA，使用Roche公司的SYBR Green Master Mix进行荧光定量PCR。PCR进行40个循环，循环参数为（94 ℃ 30 s, 54.5 ℃ 30 s, 72 ℃ 30 s）。引物如下: TCF21上游20 bp，5'-CCAGCTACATCGCCCACTTG-3'，下游23 bp，5'-CTTTCAGGTCACTCTCGGGTTTC-3'，GAPDH上游20 bp，5'-ACCACAGTCCATGCCATCAC-3'，下游20 bp，5'-TCCACCACCCTGTTGCTGTA-3'。*TCF21*基因转录水平按照2^-ΔΔCt^法通过管家基因*GAPDH*进行校正。

### 1.4 Western blot

使用细胞裂解液从各组细胞中提取细胞总蛋白。蛋白样品用12% SDS-PAGE跑胶后转膜至0.22 μm的PVDF膜上。5%牛奶室温封闭1 h，4 oC孵育TCF21（1:1, 000稀释）和GAPDH（1:5, 000稀释）一抗过夜。兔抗人多克隆抗TCF21抗体购自Abcam公司（Ab32981）。充分洗膜后室温孵育二抗1 h。再次洗膜后于暗室中显影、定影。

### 1.5 MTT

在96孔板中分别转染和处理三组细胞，分别在转染后24 h、48 h、72 h、96 h加入20 μL/孔5 mg/mL的MTT溶液；继续37 oC温箱培养4 h，小心吸尽培养液，每孔加入150 μL二甲基亚砜；轻轻振荡溶解结晶之后上酶标仪于490 nm处检测吸光度（*A*）值，实验重复3次，绘制生长曲线。

### 1.6 迁移实验

收集转染后72 h的三组细胞并计数，用1640培养基调整细胞密度至1×10^5^个/mL。将200 μL细胞悬液接种至Transwell小室的上室，下室添加700 μL含20%胎牛血清的1640培养基。温箱培养12 h后用棉签擦去上室表面的细胞。下室表面的细胞使用苏木素染色2 min。显微镜下随机选取5个视野观察计数，计算平均穿膜细胞数。每组实验重复三次。

### 1.7 流式细胞术

收集转染后72 h的三组细胞，计数细胞，每管取5×10^5^个细胞，然后按照凋亡检测试剂盒说明书步骤操作如下：每管加50 μL Binding Buffer和5 μL 7-AAD的混合液，室温避光反应15 min。依次加入450 μL Binding Buffer和1 μL Annexin V-PE，室温避光反应15 min后上流式细胞仪检测。每组重复3次。Apoptosis Detection Kit购自凯基生物有限公司。

### 1.8 统计学处理

计数资料两组间比较使用卡方检验。计量资料实验数据以Mean±SD表示，两组间均数比较采用*t*检验，多组均数比较采用单因素方差分析，两两比较采用*LSD*法。使用SPSS 17.0统计软件进行统计分析，以*P* < 0.05为差异有统计学意义。

## 2 结果

### 2.1 Lenti-TCF21成功转染细胞

为了检测慢病毒转染细胞的转染效率，我们用荧光显微镜对转染细胞进观察计数。过表达组和空白载体组均见有绿色荧光蛋白表达，且在转染72 h后观察到荧光强度最强，平均转染效率为85%（图 1）。

**1:**
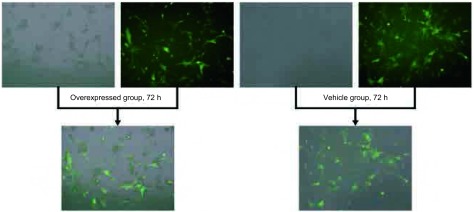
TCF21在肺癌细胞A549中的表达 The overexpression of TCF21 in A549 lung cancer cells

### 2.2 荧光定量PCR检测*TCF21*基因mRNA的表达水平

为了检测TCF21是否过表达成功，我们对转染后72 h的细胞进行了更精确的荧光定量PCR检测。结果显示过表达组TCF21 mRNA表达量远远超过未处理组和空白载体组的TCF21 mRNA表达量（2, 705.59±231.22 *vs* 1.00±0.31, 1.43±0.73）（*P* < 0.05）（图 2A）。

**2:**
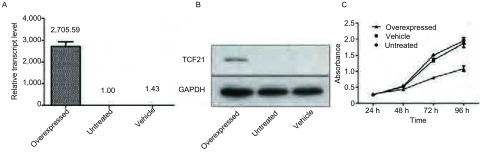
TCF21在肺癌细胞A549中的表达及对细胞增殖的影响。A：荧光定量PCR检测TCF21 mRNA在各组中的表达量；B：Western blot检测TCF21蛋白在各组中的表达量；C：MTT检测过表达TCF21对各组细胞增殖的影响 The overexpression of TCF21 in A549 lung cancer cells and its influence on cell proliferation. A: Fluorescence quantitative polymerase chain reaction was used to analyze the expression of TCF21 mRNA; B: Western blot was used to analyze the expression of TCF21 protein; C: Proliferation assay were applied to test the impact of TCF21 expression on the cell proliferation of A549 after transfection

### 2.3 Western blot检测TCF21蛋白的表达水平

为进一步检测TCF21蛋白是否表达成功，我们采用Western blot技术检测三组细胞中TCF21蛋白表达量。结果显示相对于未处理组，过表达组TCF21表达明显增高，而空白载体组和未处理组未见明显TCF21蛋白表达。这与荧光定量PCR的检测结果一致，更进一步说明A549细胞中过表达了TCF21蛋白（图 2B）。

### 2.4 过表达TCF21对A549细胞增殖的影响

对转染后的三组细胞采用MTT法进行了细胞增殖活性实验。实验结果显示：高表达组与空白载体组和未处理组比较，在24 h的检测结果显示三组间无统计学差异（*P* > 0.05）。而在48 h、72 h、96 h的检测结果显示实验组OD值明显低于空白载体组和未处理组（0.44±0.02 *vs* 0.52±0.03, 0.54±0.04; 0.80±0.05 *vs* 1.35±0.06, 1.50±0.05; 1.08±0.08 *vs* 1.87±0.13, 1.96±0.10）（*P* < 0.05），而后两者之间无明显差异。说明TCF21能够在48 h以后明显抑制肺癌细胞A549的增殖（图 2C）。

### 2.5 过表达TCF21对A549细胞迁移能力的影响

我们对三组细胞进行了Transwell细胞迁移实验。结果显示实验组细胞迁移率要明显低于空白载体组和未处理组（24.53±4.92 *vs* 54.53±8.57, 58.27±9.10）（*P* < 0.05）（图 3），而后两者之间无明显差异。这一结果说明TCF21的过表达能够明显抑制肺癌细胞A549的迁移。

**3:**
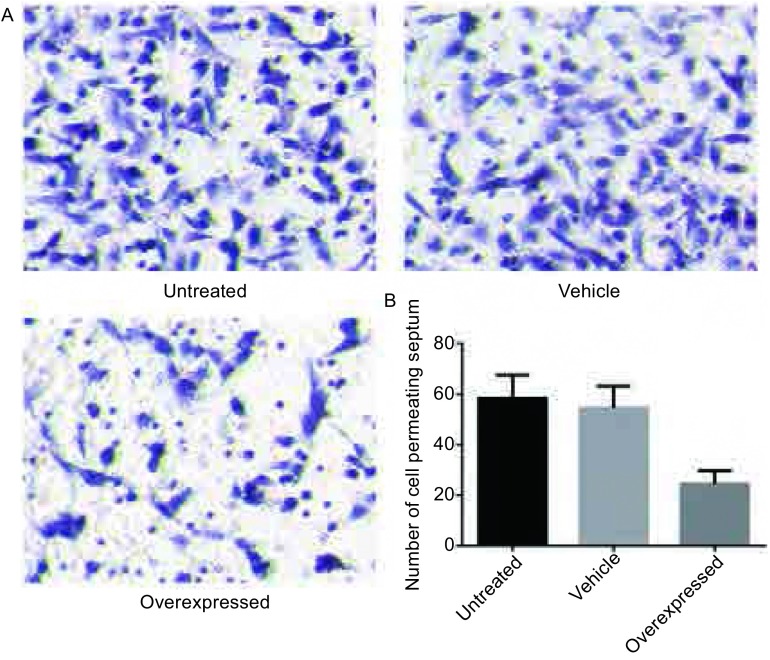
TCF21过表达对肺癌细胞A549迁移能力的影响。A：Transwell检测三组细胞迁移能力。B：三组细胞的穿膜细胞数统计图 The impact of TCF21 overexpression on the cell migration of lung cancer cell A549. A: Transwell were applied to test the impact of TCF21 expression on the cell migration of A549 after transfection. B: Statistical figure of the numbers of cell permeating septum in three groups

### 2.6 过表达TCF21对A549凋亡率的影响

在转染后72 h使用流式细胞仪对三组细胞进行了细胞凋亡分析。结果显示高表达TCF21蛋白的实验组较空白载体组和未处理组细胞凋亡率明显上升（16.93±1.21 *vs* 1.29±0.32, 0.76±0.39, *P* < 0.05）且为早期凋亡，而后两者之间无明显差异。这一结果说明TCF21的过表达能够促进肺癌细胞A549发生早期凋亡（图 4）。

**4:**
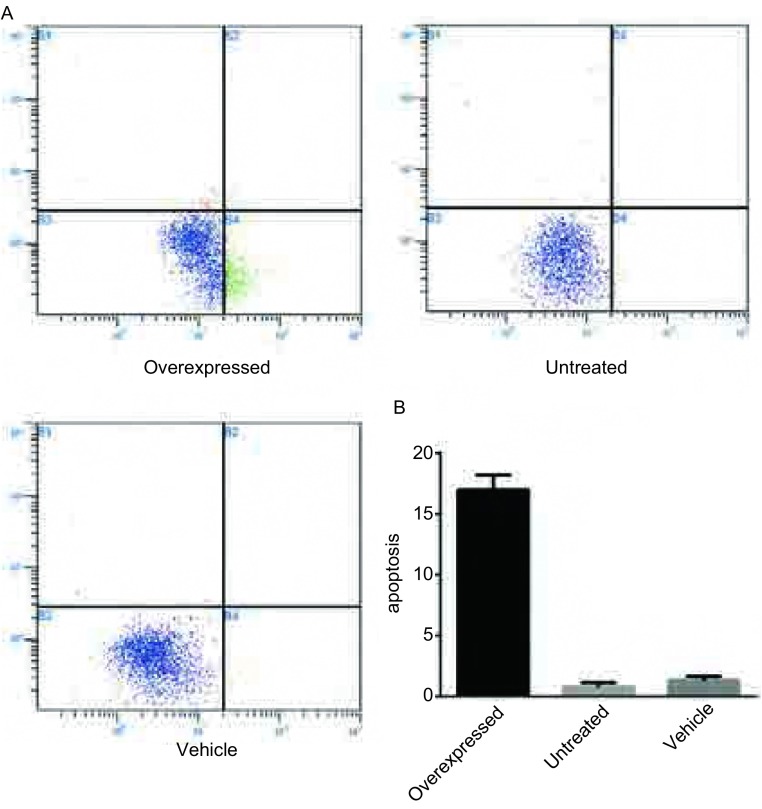
TCF21过表达对肺癌细胞A549凋亡率的影响。A：流式细胞术检测三组细胞凋亡率。B：三组细胞凋亡率统计图 The impact of TCF21 overexpression on apoptosis rate of lung cancer cell A549. A: Flow cytometry were applied to test the impact of TCF21 expression on the cell apoptosis of A549 after transfection; B: Statistical figure of apoptosis rate in three groups

## 3 讨论

研究^[17-21]^发现多种抑癌基因（如*p53*、*PTEN*、*Rb*等）在肺癌等肿瘤中存在缺失、突变或异常低表达等改变，且表达量随肿瘤恶性程度的增高而降低。*TCF21*在多种肿瘤中存在启动子区甲基化，且其在肿瘤组织中的表达量较非肿瘤组织明显降低^[11-13]^。本课题组前期关于TCF21表达和启动子区甲基化的研究也证实了这一结论^[14]^。另外有研究^[11]^显示TCF21的表达量与患者的低生存率存在明显相关性。并且*TCF21*的甲基化能够通过患者的血样检测出来^[13]^，提示TCF21可能是一个潜在的肿瘤标记物，并且检测这种标记物所需的样品容易获取，检测十分方便。

本研究在组织水平的研究基础上通过体外细胞培养进一步探索TCF21的抑癌功能，结果发现过表达TCF21能够显著抑制肺癌细胞A549生长和引起细胞凋亡率的升高。本研究在前人的基础上^[22]^有新的突破，TCF21对肺癌凋亡率的影响也部分解释了TCF21为什么能够抑制细胞增殖。同时也从侧面支持了Smith等^[22]^的结论。近期有报道^[15, 16]^称TCF21能够直接作用于*KISS1*基因，促使其表达上调，而KISS1能够间接地触发细胞凋亡^[23]^。那么TCF21是否是通过KISS1介导而实现促进凋亡的作用，这一猜想尚需实验证实。另一方面TCF21是一个转录因子，众所周知，转录因子与RNA聚合酶Ⅱ形成转录起始复合物，共同参与多种转录起始的过程^[24, 25]^。那么是否TCF21还参与KISS1以外的其他基因启动子区的调控，从而实现抑癌功能，这需要我们进一步拓展研究。本研究通过肺腺癌细胞株A549来研究*TCF21*基因的抑癌功能及其机制，问题在于A549不能够完全代表其他类型的非小细胞肺癌，体外细胞实验不能够完全模拟体内生理环境，故实验存在一定的局限性。对于本研究中*TCF21*基因过表达引起的增殖抑制和细胞凋亡率增加，为进一步确定其准确性，应该进一步检测细胞增殖和凋亡相关蛋白的表达量，在我们的后期研究中，我们会进一步完成相关蛋白的检测。这也为我们后期研究留下了空间。

本研究的结果展示了TCF21的抑癌功能，同时也提示我们可以进一步扩展探索TCF21的其他功能，包括TCF21对肺癌细胞放疗敏感性的影响，化疗敏感性的影响，对裸鼠体内成瘤能力的影响。这为我们展示了研究TCF21的巨大潜力。在我们的后期研究中我们将一方面致力于扩展*TCF21*基因在肺癌细胞中的功能研究，并在裸鼠体内做成瘤试验，以验证体外细胞实验的结论。另一方面致力于探索TCF21抑癌的具体机制，寻找其具体参与的分子调控机制。为肺癌的早期诊断和分子靶向治疗提供依据。
